# Association between fluoroquinolone resistance and MRSA genotype in Alexandria, Egypt

**DOI:** 10.1038/s41598-021-83578-2

**Published:** 2021-02-19

**Authors:** Mustafa Alseqely, Mae Newton-Foot, Amal Khalil, Mostafa El-Nakeeb, Andrew Whitelaw, Alaa Abouelfetouh

**Affiliations:** 1 Department of Microbiology and Immunology, Faculty of Pharmacy, Alexandria University, 1 Khartoum Sq., Alexandria, 21521 Egypt; 2 Division of Medical Microbiology, Stellenbosch University, Cape Town, South Africa and National Health Laboratory Service, Tygerberg Hospital, Francie van Zijl Drive, PO Box 241, Cape Town, 8000 Tygerberg South Africa; 3 Department of Microbiology and Immunology, Faculty of Pharmacy, Alalamein International University, Alalamein, Egypt

**Keywords:** Microbiology, Epidemiology, Infectious diseases

## Abstract

Antimicrobial stewardship isn’t strictly observed in most Egyptian hospitals, raising antibiotic resistance. Epidemiology of Egyptian MRSA isolates, or associations with resistance to other antibiotics remain largely unknown. We identified MRSA genotypes in Alexandria Main University Hospital (AMUH) and investigated rates of moxifloxacin resistance, an alternative MRSA treatment, among different genotypes. Antibiotic susceptibility of 72 MRSA clinical isolates collected in 2015 from AMUH was determined by disc diffusion and broth microdilution. *spa*- and Staphylococcal Cassette Chromosome *mec* (SCC*mec*) typing were performed; with multi-locus sequence typing conducted on isolates representing major genotypes. Resistance to moxifloxacin, levofloxacin and ciprofloxacin were 69%, 78% and 96%, respectively. *spa* type t037 (57%) was commonest, followed by t127 (12.5%), t267 (8%) and t688 (6%). SCC*mec* III predominated (57%), all of these were moxifloxacin resistant and 97.6% t037 (ST241). SCC*mec* IV, IV E and V represented 15%, 7% and 11% of the isolates, respectively, 79% of these were moxifloxacin susceptible and of different *spa* types. t127 (ST-1) was associated with SCC*mec* V in 56% of the isolates, mostly moxifloxacin susceptible. Moxifloxacin resistance was high, most resistant isolates belonged to t037 and SCC*mec* III, suggesting local dissemination and antibiotic pressure. We recommend caution in treating MRSA infections with moxifloxacin.

## Introduction

*Staphylococcus aureus* (*S. aureus*) is a notorious Gram positive pathogen, with clinical presentations ranging from minor skin infections to life threatening invasive infections, including pneumonia, endocarditis, osteomyelitis and bacteremia^1^. Methicillin resistant *Staphylococcus aureus* (MRSA) strains emerged in the early 1960s, and were mainly confined to hospitals (HA-MRSA)^2^. This changed in the 1990s when community acquired MRSA (CA-MRSA) emerged as a clear and present danger^3^. Since then, cases of CA-MRSA have been reported globally^4^ . In Egypt, MRSA prevalence is about 52%, a rate that is higher than most other African countries (< 50%)^5,6^.

Methicillin resistance in *S. aureus* occurs due to the expression of an additional penicillin binding protein 2a (PBP2a) with a low affinity to methicillin and most other β-lactams^7^. This ensures continuous cell wall synthesis even in the presence of the antibiotic^8^. PBP2a is encoded by a chromosomal gene *mecA* present in a mobile genetic element called the staphylococcal cassette chromosome (SCC*mec*)^9^. Over the years, methicillin resistance has driven the use of other antibiotic classes for treatment of MRSA infections, which resulted in the evolution of multi-drug resistant strains^10^. Consequently, only a few agents are still available nowadays to treat MRSA infections. While the glycopetides remain the mainstay of treatment for MRSA infections, there is limited availability of options for oral therapy. These include clindamycin, trimethoprim-sulfamethoxazole, tetracycline, and linezolid^11^. Another option, available orally, to treat MRSA infections is the fourth generation fluoroquinolone: moxifloxacin^12^. Unfortunately, resistance to these agents is being increasingly reported globally^11,13^.

Several studies have reported on moxifloxacin resistance rates^12,14–17^. However, very little is known about the level of moxifloxacin resistance among Egyptian MRSA isolates. In 2007, a study reported moxifloxacin resistance rate of 30.8% among *S. aureus* isolates from Egyptian cancer patients^18^. Another study conducted in three university hospitals in Upper Egypt found 40% of isolated MRSA from patients with hospital acquired (HA) pneumonia to be moxifloxacin resistant^19^. A more recent study, however, reported a lower level of resistance (6.3%) among *S. aureus* collected between 2015 and 2018 in Zagazig, Egypt^20^. None of these studies commented on the genotype of the studied isolates.

Bacterial typing is essential for understanding the epidemiologic and evolutionary relationships between bacterial strains, and hence for devising infection prevention strategies^21^. Moreover, MRSA genotype could affect the strain’s virulence profile with an impact on the clinical outcome making the identification of MRSA genotype important to optimize the effectiveness of MRSA therapy^22,23^.

Different molecular typing methods are currently used for *S. aureus* and MRSA typing, including SCC*mec* typing, staphylococcus protein A typing (*spa* typing), multilocus sequence typing (MLST) and pulsed-field gel electrophoresis (PFGE)^24^. Sequence based genotyping methods such as MLST and *spa* typing are better at describing the evolutionary relationships and help understand the molecular epidemiological dynamics of *S. aureus* transmission than other methods^25,26^.

In the current study, we report the prevalence and level of fluoroquinolone resistance among 72 MRSA isolates collected in 2015 from Alexandria, Egypt, as well as the associations between methicillin resistance genotype and fluoroquinolone resistance.

## Results

### Antibiotic susceptibility among the bacterial isolates

The majority of the isolates were from male patients (n = 43, 60%) and were isolated from non-ICU locations (n = 56, 78%), the median age was 35 years. We were able to categorize 56 of the isolates as being either CA or HA, of these 80% (n = 45) were HA and 20% (n = 11) were CA (Table 1).

**Table 1:** Characteristics of 72 methicillin resistant *Staphylococcus aureus* isolates obtained from Alexandria Main University Hospital.

Median age	35
Male: Female ratio	43: 28^a^
HA: CA ratio	45: 11^b^
Inpatient: ICU ratio	56: 15^c^
Specimen	Pus/tissue (n = 44)
	Blood (n = 13)
	Respiratory (n = 11)
	Urine (n = 4)
*spa* types	t037 (n = 41)
	t044 (n = 3)
	t127 (n = 9)
	t223 (n = 3)
	t267 (n = 6)
	t304 (n = 2)
	t416 (n = 1)
	t688 (n = 4)
	t786 (n = 1)
	t6978 (n = 1)
	t16221 (n = 1)
SCC*mec* types	III (n = 41)
	IV (n = 11)
	IV E (n = 5)
	V (n = 8)
	ND (n = 7)
MLST	ST-1, t127, SCC*mec* V
	ST-5, t688, SCC*mec* IV E
	ST-6, t304, SCC*mec* IV
	ST-22, t223, SCC*mec* IV E
	ST-22, t6978, SCC*mec* IV E
	ST-80, t416, SCC*mec* IV
	ST-97, t267, SCC*mec* V
	ST-239, t304, SCC*mec* III
	ST-241, t037, SCC*mec* III
	ST-1502, t044, SCC*mec* IV
	ST-4808, t267, SCC*mec* IV

a & c: gender and ward data were missing for one isolate. b: the date of admission and/or isolate collection was missing for 16 isolates.

**Figure 1:**
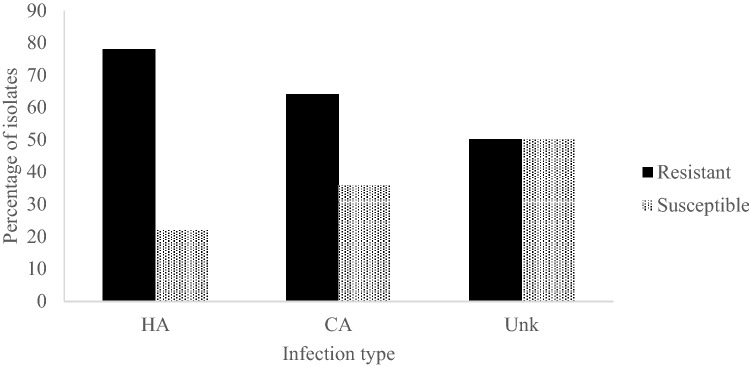
Moxifloxacin resistance among the isolates in hospital acquired (HA) and community acquired (CA) infections. Moxifloxacin resistance was seen among 78% of HA isolates, 64% of CA isolates and 50% of isolates of unknown infection type.

More isolates were susceptible to moxifloxacin (31%) than to the other tested fluoroquinolones. Moxifloxacin MICs ranged from < 0.125 to 32 μg/ml, with MIC50 and MIC90 values of 4 and 8 μg/ml, respectively. Ninety six percent of the isolates were ciprofloxacin resistant with MICs between 8 and 128 μg/ml and both MIC50 and MIC90 of 128 μg/ml. Seventy eight percent of the isolates were levofloxacin resistant with MICs ranging from 0.25 to 64 μg/ml and MIC50 and MIC90 of 16 μg/ml (Table 2). Seventy eight percent of the HA isolates were moxifloxacin resistant, compared to 64% of CA isolates (Fig. 1).

**Table 2:** Minimum inhibitory concentration (MIC) 50 and 90 of moxifloxacin, levofloxacin and ciprofloxacin against the major *spa* and SCC*mec* types represented in the isolates.

	MIC50 (µg/mL)			MIC90 (µg/mL)		
	Moxifloxacin	Levofloxacin	Ciprofloxacin	Moxifloxacin	Levofloxacin	Ciprofloxacin
***spa ***type (n)						
t037 (41)	4	16	128	8	16	128
Non-t037 (31)	2	8	32	16	16	128
All isolates (72)	4	16	128	8	16	128
**SCC** *mec* type (n)						
III (41)	4	16	128	8	16	128
IV (11)	2	8	32	2	16	128
IV E (5)	1	4	32	2	16	64
V (8)	1	8	32	16	32	64
ND (7)	2	16	64	4	16	128

### *spa* typing of the isolates

**Figure 2:**
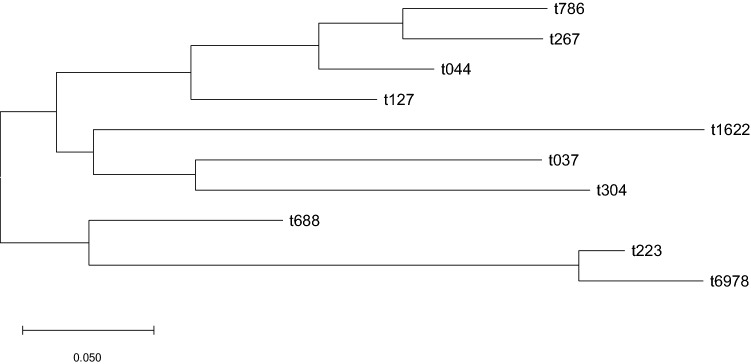
Phylogenetic tree of the spa types detected using the neighbour-joining method. The isolates were grouped in three clades, clade 1 containing isolates belonging to spa types t044, t267, t786 and t127, clade 2 containing isolates of spa t304, t037 and t1622 and clade 3 containing isolates from spa t688, t223 and t6978.

Eleven *spa* types were identified; *spa* type t037 (57%), t127 (12.5%), t267 (8%), t688 (6%), t223 (4%), t044 (4%) , t304 (3%), t786 (1%), t416 (1%), t6978 (1%), and a newly assigned *spa* type (t16221) with repeat succession (07–56-12–17-16–33-31–57-21–12) (1%). BURP analysis identified a single cluster, representing *spa* types t223 (n = 3) and t6978 (n = 1), and 8 singletons representing 93% of isolates. One *spa* type, t416 (n = 1) was excluded from the BURP analysis. Based on *spa* phylogeny, the isolates belonged to three clades: the first included *spa* types t044, t267, t786 and t127, the second clade comprised types t304, t037 and the newly identified t1622 while the rest of the identified *spa* types (t688, t223 and t6978) belonged to the third clade (Fig. 2).

### SCC*mec* typing of the isolates

**Figure 3:**
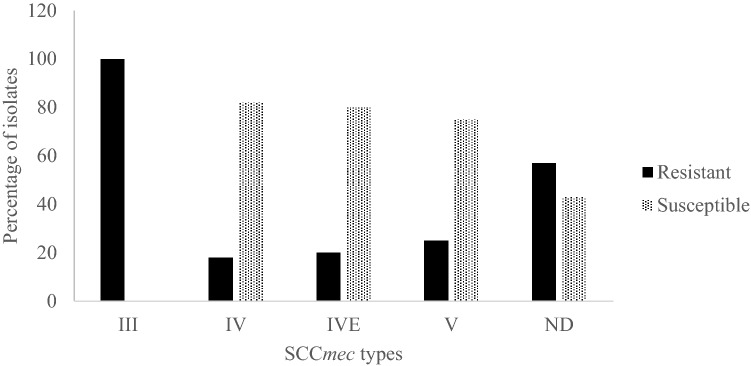
Distribution of moxifloxacin resistance among the different SCC*mec* types detected in the isolate collection. All SCC*mec* III isolates were moxifloxacin resistant whereas SCC*mec* IV, IVE and V isolates were mostly moxifloxacin susceptible.

Forty-one isolates (57%) harbored SCC*mec* type III, the vast majority (98%) of which were also *spa* type t037 (Supplementary Table S1). All of the isolates with SCC*mec* III were moxifloxacin resistant and 72.5% were from HA infections. The remaining isolates were identified as SCC*mec* types IV (15%), IVE (7%), V (11%) and an unidentified group (10%) and were mainly moxifloxacin susceptible (Fig. 3).

**Figure 4:**
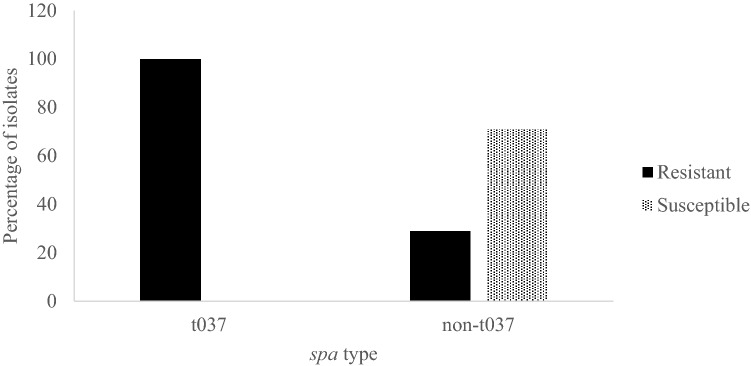
Distribution of moxifloxacin resistance among the *spa* types detected in the isolate collection. All isolates belonging to spa type t037 were moxifloxacin resistant. Isolates belonging to all other spa types were mostly (71%) moxifloxacin susceptible.

Isolates belonging to *spa* type t127 were predominantly moxifloxacin susceptible (78%). Sixty seven percent of *spa* type t267 isolates had SCC*mec* type IV, and were moxifloxacin susceptible. Fifty six percent (n = 5) of isolates belonging to *spa* type t127 also belonged to SCC*mec* type V and all but one isolate were moxifloxacin susceptible. Moxifloxacin resistance was variable among isolates belonging to *spa* types t688, t223, t044 and t304. One isolate of *spa* type t688 was identified as SCC*mec* IVE and the remaining isolates were of unknown SCC*mec* types. All isolates of *spa* type t223 and *spa* type t044 carried SCC*mec* IVE and SCC*mec* IV, respectively. Two isolates belonged to *spa* type t304; one isolate was moxifloxacin resistant and SCC*mec* III while the other isolate was moxifloxacin susceptible and SCC*mec* IV. *spa* types t416, t6978, t786 and t16221 were identified only once and in moxifloxacin susceptible isolates (Supplementary Table S1, Figs. 3–4). The newly identified *spa* type 16,221 was associated with SCC*mec* V.

### Multi-locus sequence typing of representative isolates

**Figure 5:**
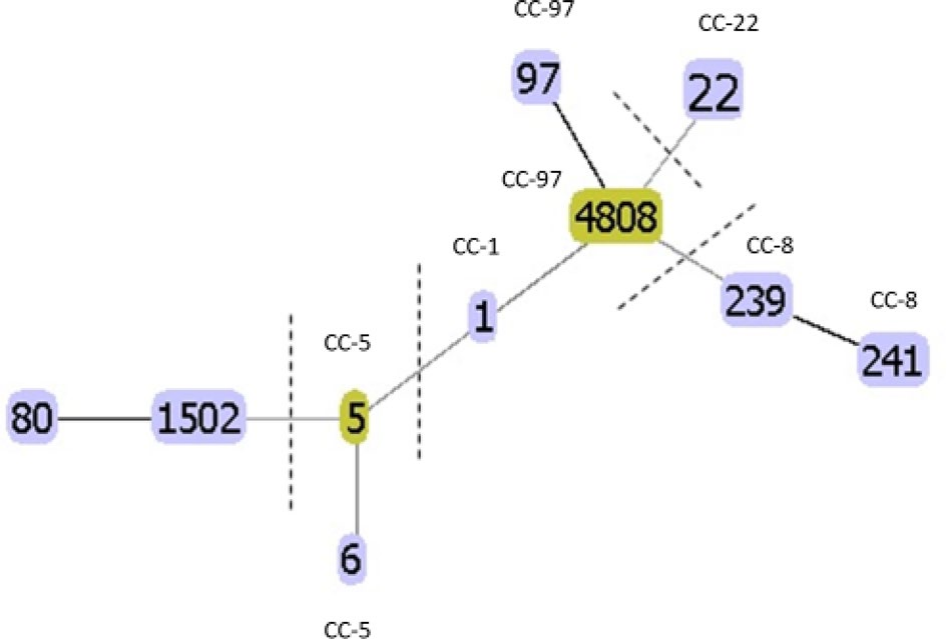
Evolutionary relationships and relatedness of MLST types using eBURST algorithm in phyloviz software. The identified MLST types belonged to four groups of ST and one singleton ST (ST-22 in CC-22). One group consisted of ST-80 and ST-1502, a second group of ST-5 and ST-6 within CC-5, a third group contained ST-1 (CC-1) and ST-97 and ST-4808 (CC-97) and a fourth group of ST-239 and ST-241 (CC-8).

Nine MLST profiles (ST-22, ST-1, ST-5, ST-6, ST-80, ST-97, ST-239, ST-241, ST-1502) were identified in addition to a new profile (3-1-1-1-1-99-3) which was assigned as MLST ST-4808. The MLST phylogenetic tree showed that the identified MLST types were related to each other within four groups; ST-239 and ST-241 were single locus variants (SLV) and moxifloxacin resistant, and were grouped under the same clonal complex (CC-8). Both ST-80 and ST-1502 had no clonal complex, yet only ST-1502 was moxifloxacin resistant. The novel type, ST-4808 was a SLV of ST-97, both of which belonging to CC-97 and a triple locus variant (TLV) of ST-1, and all were moxifloxacin susceptible. ST-5 and ST-6 were in one group (CC-5) with double locus variant (DLV) relation, both represented by isolates that were moxifloxacin susceptible. On the other hand, ST-22 was identified as a singleton with no relation to other types and was represented by a moxifloxacin susceptible isolate (Fig. 5).

## Discussion

MRSA remains a major causative agent of infections on a global scale, with worse outcomes relative to methicillin susceptible infections^27,28^. The prevalence of MRSA in Egypt is high (52%) compared to other North African and Mediterranean countries such as Morocco (19%), Libya (31%), Algeria and Tunisia (45%)^6^. In this scenario, the treatment of infections caused by MRSA requires the use of an alternative agent such as moxifloxacin^12^, that is readily available on the Egyptian market. Few reports have described the levels of moxifloxacin resistance among Egyptian *S. aureus*^18–20^. Tackling moxifloxacin resistance in Egypt requires a better understanding of the molecular lineages of Egyptian MRSA clones^21^. In this study, moxifloxacin resistance among 72 Egyptian MRSA clinical isolates was determined relative to their genotype.

Rates of resistance to fluoroquinolones were higher than previously reported^29,30^, and although moxifloxacin had the highest level of *in-vitro* susceptibility, sixty nine percent of the isolates were moxifloxacin resistant. SCC*mec* type III was the most common SCC*mec* type and almost always associated with *spa* type t037 (ST-241) and moxifloxacin resistance. This is not surprising as the *spa* t037/SCC*mec* III clone is a predominant clone in Africa^25^. The association between *spa* t037/SCC*mec* III/ST-241 and moxifloxacin resistance seen among the studied isolates is in agreement with previous findings that SCC*mec* III isolates showed 89% resistance to ciprofloxacin^31^. Moreover, a previous study described a *spa* t037/SCC*mec* III/ST-241 clone that was moxifloxacin resistant among isolates from Nigeria^32^. Almost 70% of the SCC*mec* type III isolates in the current study were HA-MRSA, in accordance with previous reports that HA-MRSA strains mainly belong to SCC*mec* types I, II and III, while CA-MRSA strains are mainly of SCC*mec* types IV and V^33–35^. In the current study, isolates belonging to SCC*mec* types IV, IVE and V were found among HA-MRSA as well as CA MRSA infections. Upon isolate collection, data on prior hospitalization was not obtained which could have changed the classification as HA or CA and might explain the discordance. However the association between SCC*mec* type and community- or hospital onset of infection is also less clear cut, possibly due to spread of hospital strains into the community and vice versa. In addition, the limited number of CA MRSA isolates in the current study makes it harder to draw solid conclusions.

The moxifloxacin resistant ST-241-III/t037 clone might represent the majority of the tested isolates as *spa* type t037 and SCC*mec* III were the most predominant types. This agrees with a study that showed that the major clonal complex causing HA-MRSA in Africa was ST-239/ST-241- III^36^. However, to the best of the authors’ knowledge this is the first report of the moxifloxacin resistant ST-239-III/t304 clone.

The majority of the isolates belonging to SCC*mec* types IV, IVE and V were susceptible to moxifloxacin with resistance rates of 18%, 20% and 25%, respectively. This concurs with Kilic et al. who showed levofloxacin resistance of 16.8% among SCC*mec* type IV isolates^37^. The moxifloxacin susceptible ST-1-V/t127 MRSA clone has been reported among human isolates in a European study, yet the porcine MRSA isolates from the same study were 95% resistant to ciprofloxacin^38^. The ST-80-IV/t416, ST-97-V/t267, ST-5-IVE/t688, ST-22-IVE/t223 and ST-22-IVE/t6978 clones reported here were all moxifloxacin susceptible. ST-97-V/t267 was previously detected in a ciprofloxacin susceptible bovine isolate from Italy^39^, the current study represents the first detection of this clone in Egypt from humans. ST-4808 is a SLV from ST-97 and the moxifloxacin susceptible clone ST-4808-IV/t267 is a novel clone firstly identified in Egypt from a human specimen in the present work. The ST-5-IVE/t688 is another new clone reported in the current study.

In conclusion, there were five new MRSA clones identified in this study; ST-239-III/t304, ST-1502-IV/t044, ST-4808-IV/t267, ST-22-IVE/t223 and ST-22-IVE/t6978 in addition to ST-97-V/t267 clone that was reported for the first time among human MRSA isolates. Resistance to fluoroquinolones was common (69% resistance to moxifloxacin), and appears to be driven by the predominant *spa* t037/SCC*mec* III clone which is moxifloxacin resistant. This may point to a potential clonal dissemination of this strain within hospitals. *spa* type t16221 and MLST ST-4808 type were newly identified in the present study, and were both moxifloxacin susceptible. The high rates of moxifloxacin resistance detected among the isolates calls for stricter implementation of antimicrobial stewardship guidelines and infection control practices among Egyptian hospitals.

## Materials and methods

### Sample collection and identification

Seventy two MRSA isolates collected from the Medical Microbiology laboratory at Alexandria Main University Hospital (AMUH) between September and December 2015 were included in the study. AMUH is the largest teaching hospital in northern Egypt with four satellite hospitals and a total capacity of 3500 beds. The isolates represented all non-duplicate MRSA isolates obtained from different clinical specimens, including pus, blood, sputum, urine, tissue, aspirate and broncho-alveolar lavage (BAL). The identity of the isolates was confirmed using Gram staining, bacterial growth and fermentation of mannitol salt agar, growth on DNase agar and slide coagulase testing using Dryspot Staphytect Plus (Oxoid Ltd, England)^40^. The isolates were classified as obtained from HA versus CA infections based on a 48 h window between admission and specimen collection.

### Antibiotic susceptibility testing

Methicillin resistance was confirmed by cefoxitin disc diffusion testing (Oxoid Ltd, England), and susceptibility of the isolates to moxifloxacin, ciprofloxacin and levofloxacin was determined using disc diffusion and confirmed by Minimum Inhibitory Concentration (MIC) determination using a broth microdilution method^41^. All susceptibility tests were performed and interpreted according to the 2015 Clinical and Laboratory Standards Institute (CLSI) guidelines^41^.

### SCC*mec* typing

DNA was extracted by boiling a suspension of colonies in 300 µl sterile distilled water for 30 min at 95 °C, followed by immediate cooling at -20 °C for 30 min. The suspension was centrifuged at 16,000 × *g* for 10 min and the supernatant was used in subsequent polymerase chain reactions (PCR)^42^. SCC*mec* typing was performed by multiplex PCR according to the protocol published by Milheirico et al.^43^. DNA extracts from six reference strains (Strains BAA-38^43^, BAA-1681^44^, BAA-39^43^, BAA-1680^45^, WIS(WBG8318)^46^ and BAA-42^43^) representing six SCC*mec* types (I, II, III, IV, V and VI) were included in each PCR reaction.

### *spa* typing

The polymorphic X region of *spa* gene was amplified according to Harmsen et al*.*^47^ and the PCR products were Sanger sequenced using the ABI 3130XL Genetic analyzer (Inqaba Biotechnologies, South Africa;). Chromatograph sequence files were processed using the BioEdit Sequence Alignment software for creation of the consensus sequence. Sequence analysis was performed using spatyper online (http://spatyper.fortinbras.us) and/or the Ridom StaphType software (Munster, Germany) and *spa* clonal complexes (*spa*-CC) were allocated using the ‘based upon repeat pattern’ (BURP) algorithm that is implemented within the software^47,48^. *spa* types that were too short to presume ancestry (< 5 repeats within the hypervariable Xr region of the *spa* gene) were excluded from the BURP analysis. A phylogenetic tree of the identified *spa* types was constructed using the Molecular Evolutionary Genetic Analysis X software (MEGA X)^49^ using the neighbour-joining method^50^. The new spa type was submitted to the Ridom spaServer under accession number 174278^51^.

### MLST

Eleven isolates representing the most common *spa* and SCC*mec* types were selected for MLST typing according to Enright et al.^52^. Briefly, seven housekeeping genes were targeted using their specific primers. The PCR products were Sanger sequenced and the consensus sequence of each gene as generated by the BioEdit Sequence Alignment software was identified as a specific allele type. The loci were combined and identified as ST types using PubMLST online (https://pubmlst.org/saureus/)^53^. A phylogenetic tree was constructed using PHYLOViZ software (http://www.phyloviz.net/) by UPGMA and eBURST^54^ to investigate the evolutionary history of the identified MLST types and the degree of relatedness. The eBURST algorithm method was applied using eBURST v3 online tool (http://eburst.mlst.net/default.asp).

## Supplementary Information

Supplementary Information.

## Data Availability

The datasets used and/or analyzed during the current study are available from the corresponding author on reasonable request.

## Abbreviations

AMUH: Alexandria Main University Hospital
BAL: Broncho Alveolar Lavage
CA: Community Acquired
CC: Clonal Complex
CLSI: Clinical and Laboratory Standards Institute
DLV: Double Locus Variant
DNA: Deoxyribo Nucleic Acid
HA: Hospital Acquired
MIC: Minimum Inhibitory Concentration
Min: Minute
MLST: Multilocus sequence typing
MOX: Moxifloxacin
MRSA: Methicillin Resistant *Staphylococcus aureus*
PBP2a: Penicillin Binding Protein 2a
PCR: Polymerase Chain Reaction
PFGE: Pulsed Field Gel Electrophoresis
S: Second
SCC*mec*: Staphylococcal cassette chromosome *mec*
SLV: Single Locus Variant
*spa*: Staphylococcus protein A
TLV: Triple Locus Variant
UPGMA: Unweighted Pair Group Method with Arithmetic Mean method
