# Supplementary material for: Association between fluoroquinolone resistance and MRSA genotype in Alexandria, Egypt
Source: Sci Rep. 2021 Feb 19;11:4253. doi: 10.1038/s41598-021-83578-2 (PMC7896087; doi:10.1038/s41598-021-83578-2)
Supplement: Supplementary file 1 — Supplementary Information. [file 41598_2021_83578_MOESM1_ESM.pdf]

Title: Association between fluoroquinolone resistance and MRSA genotype in Alexandria, Egypt.

Author names: Mustafa Alseqely, Mae Newton-Foot, Amal Khalil, Mostafa El-Nakeeb, Andrew Whitelaw, Alaa Abouelfetouh*

Supplementary Table S1: Detailed characteristics of 72 methicillin resistant *Staphylococcus aureus* isolates obtained from Alexandria Main University Hospital.

Isolate number	Patient's gender	Patient's age (Years)	Unit	Clinical specimen	Type pf infection	*spa* type	SCC*mec* type	MLST
3	Female	46	ICU	BAL	HA	t037	III	ST-241
4	Male	32	Inpatient	Pus	HA	t037	III	ND
10	Male	3	Inpatient	Pus	HA	t037	III	ND
13	Female	50	ICU	BAL	HA	t037	III	ND
14	Male	19	Inpatient	Pus	HA	t037	III	ND
22	Female	40	Inpatient	Pus	HA	t037	III	ND
23	Male	19	Inpatient	Pus	HA	t037	III	ND
27	Female	1	Inpatient	Pus	HA	t037	III	ND
29	Male	54	Inpatient	Urine	NA	t037	III	ND
33	Male	72	Inpatient	Pus	NA	t037	III	ND
37	Female	27	Inpatient	Pus	NA	t037	III	ND
44	Male	1	Inpatient	Pus	NA	t037	III	ND
45	Female	45	Inpatient	Pus	HA	t037	III	ND
47	Male	68	Inpatient	Pus	CA	t037	III	ND
48	Male	1	Inpatient	Pus	HA	t037	III	ND
50	Male	58	Inpatient	Urine	CA	t037	ND	ND
53	Female	40	Inpatient	Pus	NA	t037	III	ND
57	Female	<1 [1]	Inpatient	Pus	HA	t037	III	ND
59	Female	16	Inpatient	Sputum	NA	t037	III	ND
62	Male	59	ICU	MiniBAL	HA	t037	III	ND
63	Male	52	Inpatient	Pus	HA	t037	III	ND
64	Female	36	Inpatient	Pus	HA	t037	III	ND
65	Female	50	ICU	MiniBAL	HA	t037	III	ND
66	Female	25	Inpatient	Pus	HA	t037	III	ND
68	Male	45	Inpatient	Pus	HA	t037	III	ND

75	Male	31	Inpatient	Pus	HA	t037	III	ND
76	Male	15	Inpatient	Pus	CA	t037	III	ND
78	Female	62	ICU	BAL	CA	t037	III	ND
79	Female	25	ICU	Blood	HA	t037	III	ND
83	Female	55	Inpatient	Sputum	HA	t037	III	ND
90	Male	52	Inpatient	Pus	HA	t037	III	ND
91	Female	18	Inpatient	Tissue	HA	t037	III	ND
95	Male	72	Inpatient	Pus	CA	t037	III	ND
96	Male	63	Inpatient	Pus	HA	t037	III	ND
97	Male	33	Inpatient	Pus	HA	t037	III	ND
100	Male	34	ICU	Pus	HA	t037	III	ND
101	Male	52	Inpatient	Pus	HA	t037	III	ND
102	Female	18	Inpatient	Blood	HA	t037	III	ND
106	Male	44	ICU	Pus	HA	t037	III	ND
107	Male	12	ICU	BAL	HA	t037	III	ND
108	Male	54	ICU	BAL	CA	t037	III	ND
19	Female	4	Inpatient	Pus	HA	t044	IV	ST-1502
21	Male	18	Inpatient	Blood	HA	t044	IV	ND
46	Male	23	Emergency	Blood	CA	t044	IV	ND
1	Male	30	ICU	BAL	HA	t127	V	ST-1
20	Female	25	Inpatient	Blood	HA	t127	ND	ND
51	Male	60	ICU	Pus	HA	t127	IV	ND
52	Female	50	Inpatient	Pus	NA	t127	IV	ND
70	Male	33	Inpatient	Blood	HA	t127	V	ND
73	Male	58	ICU	Blood	CA	t127	V	ND
81	NA	NA	Inpatient	Pus	NA	t127	V	ND
82	Female	62	Inpatient	Pus	HA	t127	V	ND
103	Male	24	Inpatient	Pus	HA	t127	ND	ND
17	Female	35	Inpatient	Blood	NA	t16221	V	ND
5	Male	46	Inpatient	Blood	HA	t223	IV E	ND
7	Male	38	Inpatient	Pus	HA	t223	IV E	ND
18	Male	1.5	Inpatient	Pus	HA	t223	IV E	ST-22
6	Male	20	Inpatient	Aspirate	HA	t267	IV	ND

8	Male	50	ICU	Blood	CA	t267	IV	ND
11	Male	1	Inpatient	Pus	HA	t267	IV	ST-4808
35	Female	50	Inpatient	Pus	NA	t267	V	ND
36	Male	32	Inpatient	Blood	NA	t267	V	ST-97
39	Female	41	Inpatient	Aspirate	NA	t267	IV	ND
30	Female	42	Inpatient	Blood	NA	t304	IV	ST-6
31	Male	54	Inpatient	Pus	NA	t304	III	ST-239
2	Male	35	Inpatient	Blood	CA	t416	IV	ST-80
28	Female	25	ICU	MiniBAL	NA	t688	IV E	ST-5
74	Female	31	Inpatient	Pus	CA	t688	ND	ND
80	Female	62	Inpatient	Pus	HA	t688	ND	ND
84	Male	20	Inpatient	Urine	HA	t688	ND	ND
32	Male	<1 [2]	Inpatient	Pus	NA	t6978	IV E	ST-22
54	Male	30	Inpatient	Urine	HA	t786	ND	ND

[1] 9 months, [2] 6 months.

NA: Not available, ICU: Intensive care unit, BAL: Bronchoalveolar lavage, HA: Hospital acquired, CA: Community acquired, ND: Not determined.
